# Assessment of Skeletal Tumor Load in Metastasized Castration-Resistant Prostate Cancer Patients: A Review of Available Methods and an Overview on Future Perspectives

**DOI:** 10.3390/bioengineering5030058

**Published:** 2018-07-28

**Authors:** Francesco Fiz, Helmut Dittman, Cristina Campi, Silvia Morbelli, Cecilia Marini, Massimo Brignone, Matteo Bauckneht, Roberta Piva, Anna Maria Massone, Michele Piana, Gianmario Sambuceti, Christian la Fougère

**Affiliations:** 1 Nuclear Medicine Unit, Department of Radiology, Uni-Klinikum Tübingen, 72076 Tübingen, Germany; Helmut.Dittmann@med.uni-tuebingen.de (H.D.); christian.lafougere@uni-tuebingen.de (C.l.F.); 2 Department of Internal Medicine, University of Genoa, 16132 Genoa, Italy; 3 Nuclear Medicine Unit, Department of Medicine-DIMED, University Hospital of Padua, 35128 Padua, Italy; cristina.campi@gmail.com; 4 Nuclear Medicine Unit, Department of Health Sciences, University of Genoa, 16132 Genoa, Italy; silviadaniela.morbelli@hsanmartino.it (S.M.); bauckneht@yahoo.com (M.B.); pivaroberta@hotmail.it (R.P.); sambuceti@unige.it (G.S.); 5 National Council of Research-IBFM, 16132 Genoa, Italy; c1570@unige.it; 6 Department of Engineering, University of Genoa, Pole of Savona, 17100 Savona, Italy; massimo.brignone@unige.it; 7 National Council of Research-SPIN, 16152 Genoa, Italy; annamaria.massone@cnr.it (A.M.M.); piana@dima.unige.it (M.P.); 8 Department of Mathematics, University of Genoa, 16146 Genoa, Italy

**Keywords:** mCRPC, computational analysis, bone metastases, bone scan, PET-CT

## Abstract

Metastasized castration-resistant prostate cancer (mCRPC), is the most advanced form of prostate neoplasia, where massive spread to the skeletal tissue is frequent. Patients with this condition are benefiting from an increasing number of treatment options. However, assessing tumor response in patients with multiple localizations might be challenging. For this reason, many computational approaches have been developed in the last decades to quantify the skeletal tumor burden and treatment response. In this review, we analyzed the progressive development and diffusion of such approaches. A computerized literature search of the PubMed/Medline was conducted, including articles between January 2008 and March 2018. The search was expanded by manually reviewing the reference list of the chosen articles. Thirty-five studies were identified. The number of eligible studies greatly increased over time. Studies could be categorized in the following categories: automated analysis of 2D scans, SUV-based thresholding, hybrid CT- and SUV-based thresholding, and MRI-based thresholding. All methods are discussed in detail. Automated analysis of bone tumor burden in mCRPC is a growing field of research; when choosing the appropriate method of analysis, it is important to consider the possible advantages as well as the limitations thoroughly.

## 1. Introduction

Prostate cancer is the most common non-cutaneous neoplasia in western countries, affecting up to one in five individuals, with incidence increasing with age [1]. Depending on initial staging, a variable percentage of these patients will experience progression to castration-resistant prostate cancer, defined as evidence of rising tumor marker in spite of androgen blockade [2]. Under these conditions, metastastization to the skeletal system frequently occurs; this process can significantly contribute to morbidity and mortality [3]. Once considered as a harbinger of terminal disease, today bone metastases can be treated with a variety of modalities, including anti-androgen, cytotoxic, immune, and radioisotope therapy [2,4,5,6,7]. Owing to the new therapeutic modalities, survival of mCRPC patients has improved significantly [8]. However, skeletal metabolic alterations associated with bone metastases remain the principal prognostic factor [9]. The presence and extension of bone metastases are easily investigated with imaging methods [10]: these techniques include the identification of increased mineral density (at X-ray computed tomography), of bone turnover (afforded by bone scan or by fluoride PET/CT), as well as the characterization of the prostate-specific transmembrane antigen using PSMA-PET. While these methods can identify the characteristics associated with the presence of bone metastases, an accurate estimation of the total tumor burden is challenging, especially in patients with extensive metastastization. An exact quantification of this parameter, expressed as total volume of metastatic tissue or as a percentage of normal trabecular bone occupied by metastases, could help in prognostic stratification as well as in monitoring the therapeutic effectiveness. Over the course of the last decade, many attempts have been carried out to estimate this parameter by applying a computational algorithm to the clinical images (whether scintigraphic or X-ray based). In this review, we are going to analyze the computational methods currently available or in development, to identify the advantages and the disadvantage of each one of them, in order to clarify which of these methods is the most promising for current mCRPC patient care.

## 2. Methods

### 2.1. Search Strategy

A computerized literature search of the PubMed/Medline was conducted. A search algorithm, based on a combination of these terms: “metastatic castration-resistant prostate cancer” OR “metastatic CRPC” AND “computational analysis” OR “bone automated analysis” OR “bone tumor burden” OR “bone tumor volume”. This search comprised articles published in English in the last decade, i.e., between 2008 and 2018. This choice was motivated by the need to describe the current development in computational analysis and to focus on the currently used methods, also considering the current and constantly evolving computational capabilities of the actual devices.

The search was expanded by manually reviewing the reference list of the chosen articles.

### 2.2. Study Selection

Studies or study subset, which investigated the role of computational/automated analysis of CT, SPECT/CT, or PET/CT images in identifying and quantifying bone metastases from CRPC, were included. The following articles were excluded: (I) articles outside the field of interest of the current review; (II) editorials, letters, or conference proceedings; (III) case reports; (IV) phantom, simulation, and/or preclinical studies. Titles and abstract were first screened; in case of unequivocal ineligibility the article was rejected. The full text of the articles that passed this screening process was then assessed to determine its eligibility. In these selected papers, a thorough evaluation of the references was conducted, to identify potential further studies. 

**Figure 1:**
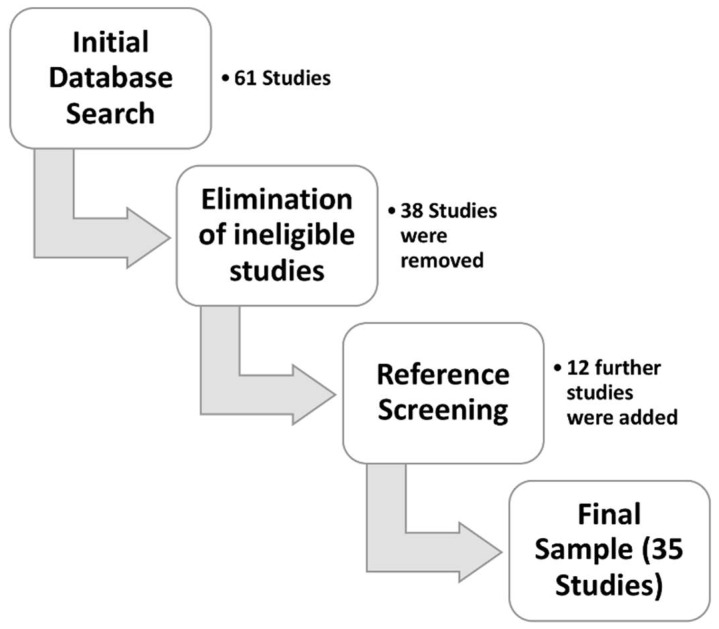
Study selection workflow. Here are detailed the steps required to construct the study database of the present study.

The selection process is outlined in Figure 1.

### 2.3. Article Categorization

For each selected article, information was collected about the following parameters: basic information (journal, year of publication, author names and country of origin, study type); technical characteristics (type of device used, type of performed analysis, planar or 3D imaging, type of tracer etc.), and finally patients’ data (number of enrolled patients, mean age, average Gleason and prevalence of “high risk” patients, mean PSA level).

## 3. Results and Discussion

### 3.1. General Parameter

**Figure 2:**
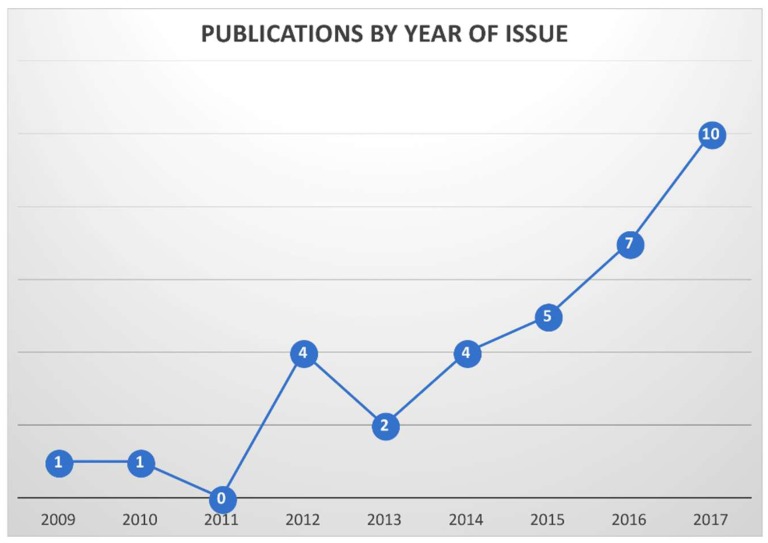
Temporal distribution of the selected studies. Nearly half of the computational studies were published in the last two years.

In total, 35 studies were identified. The main studies parameters are listed in Table 1 and in Figure 2. Twenty-five studies (72%) were single-institution, nine studies (25%) were multi-institution, and one was a review [11].

The number of eligible studies increased over time, indicating a growing interest in the subject of automated analysis of tumor burden (Figure 1). Most single-institution studies (n = 9/25, 36%) originated from the United States; moreover, most of the multi-institutional studies included an American center (n = 6/9, 67%) [12,13,14,15,16,17,18,19,20,21,22]. Five studies originated from Germany [23,24,25,26,27], four from Sweden [28,29,30,31], and six from Japan [32,33,34,35,36,37]. Information about patient number, acquisition technique, type of employed isotope (when applicable) and method of computational analysis were obtainable from all studies. Seven studies (21%) did not include information about mean patients’ age; one did not specify the enrollment method. Twenty-five studies (73%) provided the mean PSA level at the time of scan; only seven (21%) allowed obtaining information about the “high risk” status.

Mean age was very consistent across the studies, ranging from 65 to 75 years. Conversely, PSA levels were very heterogenous, probably because of different clinical protocols and disease aggressiveness. Studies conducted in the US utilized mostly 18F-NaF PET/CT, where this tracer was “re-discovered” following the spread of hybrid PET/CT imaging [46]. In Germany, most studies were focused on 68Ga-PSMA-PET/CT, as there is a long-standing history of development and theranostic use of this tracer [47]. In Sweden, studies using an artificial neural network method, applied to 2D bone scintigraphies (EXINI bone scan, EXINI Diagnostics, Lund, Sweden) prevailed [28]; a similar situation is observed in Japan, where a version of this tool, based on a custom database (BONENAVI, FUJIFILM RI Pharma, Tokyo, Japan), is marketed [32]. 

### 3.2. Types of Computational Approaches

Our literature research shows that the problem of skeletal tumor volume quantification in metastatic mCRPC has been tackled using different methods. For the purpose of the current review, we sorted these approaches in four different categories: 2D bone scan segmentation, 3D segmentation based on SUV threshold, 3D segmentation based on CT data or on CT/SUV integration, and MR-based or non-isotope-based methods. Each of these methods presents its own advantages and limitations; in the following parts we are going to discuss them in detail. An overview of the methods’ characteristics can be found in Table 2.

#### 3.2.1. Automated 2D Analysis of Bone Scans

This computational technique utilizes an automated analysis of tracer distribution, based on a neural network, to identify and quantify bone metastases on planar bone scintigraphies automatically [32,38]. It proceeds by comparing the selected scan to those present in its database to tell apart tumor-related uptakes from the physiological tracer distribution. This algorithm should in fact allow isolating the signal of metastases from normal structures (urinary bladder, kidneys, etc.). The program can also provide an estimate, the bone scan index (BSI) which represents the percentage of available intraosseous space invaded from metastases. The software application can be also re-trained with different, region-specific databases; to this date two main versions are available: the EXINI bone scan (EXINI Diagnostics, Lund, Sweden), which was calibrated with scan obtained from Swedish patients [28] and the BONENAVI system (FUJIFILM RI Pharma, Tokyo, Japan), trained with scintigraphies from Japanese individuals [32].

Over the course of years, BSI has been used to estimate the total tumor burden and to predict the patients’ clinical course. An early report from Sadik and colleagues indicated that the use of this system increased the sensitivity and the percent of agreement of the reporting physicians [28]. A later paper by Takahashi indicated that the automated method works at best with manual corrections from the reader [32]. A work by Meirelles observed how BSI is a strong predictor of survival in mCRPC patients; but so was 18F-FDG, probably as a measure of tumor de-differentiation and aggressiveness [39]. Mitusi and Dennis showed that a decrease of BSI after therapy was predictive of survival [35,40]; Reza showed that BSI changes predicted progression-free survival in patients treated with the novel anti-androgen Darolutamide [41]. Parallelly, Anand and colleagues analyzed the correlation between BSI and enzalutamide response [21]. In a paper from the group of Kaboteh, a strong association between BSI, appearance of new lesions and two-years survival was demonstrated [31]. Furthermore, a correlation was observed between BSI and diverse bone turnover markers [33]. The paper by Armstrong et al. indicated a correlation between imaging and biochemical markers as well as a strong association between bone-related imaging index and survival [22]. A further survival analysis was performed in the work by Uemura [36], where BSI was pitted against other potential survival predictors and showed a significant discriminatory power.

The paper from Miederer was the first paper investigating the correlation between BSI and insurgence of hematologic toxicity in a group of mCRPC treated with 223-RaCl_2_ [27]. Remarkably, the incidence of toxicity reported by this paper is significantly higher than the one described in the larger validation trials; this is likely to depend from the greater tumor load in his patient population. On the same line, the work by the group of Alva identified the group of patients with BSI < 5 as optimal candidates for the 223RaCl_2_ therapy, as these were more likely to be able to receive the entire course of six cycles of the bone-seeking alpha-emitter [20]. Recently, Fosbøl and co-worker confirmed the capability of BSI to predict both overall survival and hematologic toxicity in a larger series [42].

In the work from Shintawati and co-workers, BSI was evaluated on bone scan acquired at different time points after injection [34]. The index increased significantly from 2 h p.i. up to 4 h and again in the late 6 h scan; the variation was greatest in the pelvic segment. In the process, some physiological uptakes were sometimes misclassified as lesions at different time points. The authors conclude that the acquisition time should be standardized and that the neural network should be again trained with scans acquired at that specific time point.

Finally, a work by Thomas et al. compared BSI, visual analysis and 68-Ga-PSMA-PET/CT: the latter was able to detect more lesions [25]. However, no changes in clinical management would have resulted from the improved detection.

In general, the advantages linked with this method are the easiness of use, the prompt availability of an easy to interpret score, the wide diffusion of bone scintigraphy as an imaging method, and its reproducibility [34]. The disadvantages of such an approach lay in the bidimensional nature of the bone scan and in the lack of specificity of the method. In fact, there is no established uptake threshold that can distinguish benign from metastatic uptake on planar scintigraphy: for this reason, fractures, degenerative joint disease, overlapping renal uptake, benign cartilage calcifications, Paget’s disease, and many more instance could all be misclassified as tumor volume, especially when in proximity or overlapping with metastatic foci. All these instances need to be manually corrected by the operator; however, the necessary level of certainty to make such corrections can only be achieved by integrating the scan with SPECT imaging or by resorting to a co-registered/recent CT.

Moreover, if the system is trained on a specific population, it might not function properly when applied to a different one. In fact, Takahashi and co-workers reported a significant increase in specificity when using a program that was trained specifically for the local population when compared to a software application trained with a foreign one. The authors attributed this phenomenon to a difference in the average physical constitution, which causes a different photon attenuation that cannot be compensated in the absence of transmissive information.

Finally, if used to perform longitudinal evaluations, the method could be misguided by the so-called “flare” reaction, represented by a temporary an increase of activity within the bone localizations, which then is followed by a radiological response [48].

#### 3.2.2. SUV-Based Thresholding

This method is applied to PET (or possibly to quantitative SPECT) and utilizes a definite standardized uptake value (SUV) cutoff to calculate the volume of tissue with high tracer uptake, which is supposed to be expression of metastatic involvement. Many tracers were tested with this method: 18F-NaF [14,30], 11C-Choline [12,19], and 68Ga-PSMA [24]; it has the advantages of affording a 3D evaluation, thus eliminating the overlap artifacts, and to be relatively easy and speedy to employ. Moreover, whenever a co-registered CT or MR is available, the specificity of the method is significantly higher, as the operator can use the morphological data to eliminate all the uptake source that may not be related to tumor manifestations manually.

Similarly to BSI, PET-based tumor volume segmentation was used to predict the patients’ clinical outcome. The earliest work that used this approach dates to 2014, when Kwee and colleagues calculated tumor volume and activity on a series of 11C-Choline scans [12,19]. These parameters strongly correlated with PSA level and were able to predict survival at univariate survival analysis. The same group used the same index to assess treatment response (a variety of therapeutic modalities were employed) and concluded that automated analysis could be relevant in predicting therapy outcome and thus play a role in personalized medicine Later on, a group at Texas University established a SUV threshold for tumor volume in fluoride PET and used this value to predict overall survival, hematologic toxicity, and skeletal-related events [14,15,18]. In 2017, a paper by Wassberg demonstrated the repeatability of NaF-derived volumetric parameters [30]. 

A recent a large series by Schmuck, Derlin, and co-workers translated the concept of 3D segmentation onto the PSMA-based PET imaging [24]. Here, the authors demonstrated a very tight correlation between 68-Ga-PSMA based tumor volume and circulating PSA levels, moreover, volumetric parameters predicted therapy efficacy. 

Lastly, a recent work by Umeda et al. used quantitative SPECT to establish a SUV threshold, which was used to discriminate metastases from degenerative changes and to compute a “total bone uptake” as a measure of tumor burden [37]. This value was compared to BSI and resulted slightly more efficient in assessing presence of disease a therapy response. 

In PET- and SPECT-based segmentation, an accurate standardization is needed, since SUV is dependent from different factors, like image acquisition and data reconstruction, VOI definition, tracer-specific pathophysiological factors, or the kinetics of tracer distribution. Even in single-center studies, SUV can show relevant variation over the years, as the detectors’ sensitivity dwindles [49]. Besides, the adoption of a fixed threshold does not in any way ensure a full specificity of the segmented uptake (as SUV of many benign processes overlaps with the metastases-related one) [50]; increasing this threshold might result in a loss of sensitivity while decreasing it might affect specificity. In general, SUV-based analyses share the limitations of bone scan evaluations, since the risk of establishing an inadequate threshold cannot be fully eliminated. However, the 3D nature and the presence of co-registered morphological imaging can help the reader in correcting such automated evaluations. Moreover, the adoption of more specific tracers, such as 68Ga- or 18F-PSMA can increase the methods specificity when compared to bene-seeking tracers, thus allowing for a more confident application of this method.

#### 3.2.3. Hybrid CT- and SUV-Based Thresholding

In order to overcome the limitations of uptake threshold techniques, hybrid analysis techniques have been developed in the last few years [13,16,17,23,26]. These methods are applied to multi-modal imaging (PET/CT or SPECT/CT) and use the information from both the morphologic and the functional dataset to define the tumor volume accurately. The advantage of this approach is the high degree of accuracy: for instance, the methodic proposed by Bieth and colleagues [23] starts by recognizing the bone and by eliminating all the soft tissue; then, it uses a PET-based segmentation to identify the uptakes within the bone volume. As PSMA uptake in bone is considered to be very specific and all external PSMA sources have been taken out, it results that this method can provide a very accurate reading of bone tumor volume. Another approach, developed by our group on the basis of a bone marrow segmentation application [26,51,52,53,54], utilizes a CT-based adaptive threshold algorithm to discriminate between normal trabecular bone and metastases. These CT-based volumes are then translated onto the co-registered functional (PET or SPECT) images [26]. 

Using this method, the program can provide both CT- and SPECT/PET-based information, which may be correlated with clinical and laboratory parameter. In this study, we observed how metabolic intensity in trabecular bone and tumor volume are predictor of hematologic toxicity in patients treated with 223RaCl_2_ therapy.

Other hybrid methods include the one proposed by Yip [13], who employed an articulated registration to segment bone lesions on 18F-NaF-PET/CT scans. This method performed better than rigid and deformable algorithms. Harmon et al. also segmented bone lesion using an SUV threshold, after having sorted out all non-osseous tissue by using a CT-based mask [17]. Their result demonstrated how imaging-grounded metrics (including tumor burden, SUVtotal, and SUVmean) outperformed clinical parameters in predicting progression-free survival. Finally, the group led by Lindgren Belal compared two methods of hybrid segmentation (semi-automatic and manual) to BSI [29]. The three methods were equally effective in predicting overall survival.

Generally, these ‘hybrid’ methods can potentially produce more reliable results than the ones based on counts or SUV thresholding, since they start by defining the target bone region, thus excluding many confounding uptake sources (such as the urinary system). Moreover, they can produce a large quantity of potentially useful information, including morphological parameters such as density and volume as well as functional ones such as uptake intensity. They are however relatively complicated and not completely suitable to everyday practice, as the analysis is complex, requiring a certain amount of training and necessitating a relevant computational power or a long elaboration time. For these reasons, such methods are presently more appropriate for research applications.

#### 3.2.4. MR-Based and other Non-Isotopic Methods

Magnetic resonance imaging (MRI) is actually a powerful tool in detection of tumor burden in mCRPC. However, due to the long acquisition time of whole-body imaging and the concerns due to patients’ discomfort, it is only rarely used in staging and re-staging of these patients. Nonetheless, new acquisition techniques, able to speed up the scan process, and the rise of hybrid PET/MR system could signify an important role for MRI in mCRPC. Accordingly, techniques used to estimate the osseous tumor burden in MRI are in course of development. Blackledge et al. devised a semi-automatic method, in which the operator adjusted a threshold parameter to delimit the tumor localizations [43]. In this volume, a probabilistic model is applied to reduce the misclassified voxels. In the whole patient population, subjects who responded to treatment displayed a greater global apparent diffusion coefficient than non-responders.

Perez-Lopez and colleagues utilized a semi-automatic segmentation of T1 and diffusion-weighted images to calculate the tumor burden, then they compared these results with BSI [44]. Tumor volume and BSI were correlated; furthermore, tumor burden could predict survival and was associated with many clinical and radiological parameters.

Finally, Brisset and colleagues set up a complex study, in which, among other parameters, investigated CT- and MRI-related parameter with a voxel based-analysis [45]. Such parameters were able to tell apart stable from progressive disease at a longitudinal analysis.

In general, MRI-based method offer the advantage of excellent lesion contrast and the potential to detect also non-osseous localizations with a high degree of confidence. Moreover, the use of MRI can spare the patients, handling personnel, and general population from radiation exposure. However, whole-body MRI is not yet diffuse due to its relatively long acquisition time and thus, low patient throughput. Although studies on this matter are limited, it remains a promising research path.

## 4. Conclusions

The application of computational analysis methods to planar or hybrid imaging is a constantly growing field of imaging science. In the clinical scenario of metastatic CRPC, whose prevalence has been steadily increasing in recent years, it could improve patients management by allowing an accurate quantification of tumor load and produce a reliable index to test the therapeutic effectiveness of the many new treatment methods. Moreover, it has the potential to turn the everyday scan in a plentiful source of information, which can non-invasively disclose a great quantity of information on tumor biology as well as on therapy efficacy.

When choosing the most proper approach, it is important to strike a balance between striving for rapid results and avoiding inaccuracy. In this line, it appears that choosing a specific tracer (such as 68Ga-PSMA) and using all the available information (including functional and morphologic data) can be helpful in tackling this endeavor and in ensuring the best patient care.

## Figures and Tables

**Table 1:** Characteristics of the selected studies.

First Author	Year	Reference	Country	Type of Study	Pts. Number	Techinique	Tracer	Analysis	Mean Age	Mean Gleason	Mean PSA (ng/mL)	High Risk Ratio
NAKAJIMA	2017	[11]	JAPAN/SWEDEN	RW	-	-	-	REVIEW	-	-	-	-
KWEE	2014	[12]	USA	P	30	PET/CT	18F-CHOLINE	PET-BASED SEGMENTATION	73	N/A	35.1	N/A
YIP	2014	[13]	USA	NS	16	PET/CT	18F-FLUORIDE	HYBRID CT- AND PET-BASED SEGMENTATION	69	N/A	N/A	N/A
ETCHEBEHERE	2015	[14]	USA	R	42	PET/CT	18F-FLUORIDE	PET-BASED SEGMENTATION	71.7	N/A	54	64.3%
ROHREN	2015	[15]	USA	R	68	PET/CT	18F-FLUORIDE	PET-BASED SEGMENTATION	65.7	N/A	N/A	N/A
LIN	2016	[16]	USA	P	35	PET/CT	18F-FLUORIDE	HYBRID CT- AND PET-BASED SEGMENTATION	71.5	7.8	49	41%
HARMON	2017	[17]	USA	P	58	PET/CT	18F-FLUORIDE	HYBRID CT- AND PET-BASED SEGMENTATION	71	N/A	N/A	48%
ETCHEBEHERE	2016	[18]	USA/BRASIL	R	41	PET/CT	18F-FLUORIDE	PET-BASED SEGMENTATION	71	N/A	150	61.9%
LEE	2016	[19]	SOUTH KOREA/USA	P	42	PET/CT	18F-CHOLINE	PET-BASED SEGMENTATION	73	N/A	329	N/A
ALVA	2017	[20]	USA/SWEDEN	R	145	BONE SCAN	99mTc-DPD	EXINI BONE SCAN ANN	71.8	9	188.7	70%
ANAND	2016	[21]	USA/SWEDEN	R	80	BONE SCAN	99mTc-MDP	EXINI BONE SCAN ANN	71	N/A	157.5	N/A
ARMSTRONG	2014	[22]	USA/SWEDEN	R	85	BONE SCAN	NOT SPECIFIED *	EXINI BONE SCAN ANN	N/A	N/A	N/A	N/A
BIETH	2017	[23]	GERMANY	R	45	PET/CT	68-Ga-PSMA	HYBRID CT- AND PET-BASED SEGMENTATION	71	N/A	43	N/A
SCHMUCK	2017	[24]	GERMANY	R	101	PET/CT	68-Ga-PSMA	PET-BASED SEGMENTATION	69.1	7 ***	4.1	N/A
THOMAS	2017	[25]	GERMANY	R	30	BONE SCAN AND PET/CT	99mTc-MPD AND 68-Ga-PSMA	EXINI BONE SCAN ANN; VISUAL ANALYSIS	N/A	N/A	N/A	N/A
FIZ	2017	[26]	GERMANY/ITALY	R	47	BONE SPECT/CT	99mTc-DPD	CT-BASED SEGMENTATION	69.5	8	788	68%
MIEDERER	2015	[27]	GERMANY	R	14 **	BONE SCAN	99mTc-DPD	EXINI BONE SCAN ANN	71	N/A	N/A	N/A
SADIK	2009	[28]	SWEDEN	R	41	BONE SCAN	99mTc-MPD	EXINI BONE SCAN ANN	65	N/A	N/A	N/A
LINDGREN BELAI	2017	[29]	SWEDEN	R	48	BONE SCAN AND PET/CT	99mTc-HPD AND 18-F-FLUORIDE	HYBRID CT- AND PET-BASED SEGMENTATION; EXINI BONE SCAN ANN	73	7.7	374	N/A
WASSBERG	2017	[30]	SWEDEN	P	10	PET/CT	18F-FLUORIDE	PET-BASED SEGMENTATION	74.6	8.1	208.5	50%
KABOTEH	2013	[31]	SWEDEN	R	266	BONE SCAN	99mTc-MDP	EXINI BONE SCAN ANN	76	N/A	N/A	N/A
TAKAHASHI	2012	[32]	JAPAN	R	158	BONE SCAN	99mTc-MPD	BONENAVI BONE SCAN ANN	69.5	N/A	148	N/A
WAKABAYASHI	2013	[33]	JAPAN	R	52	BONE SCAN	99mTc-MPD	BONENAVI BONE SCAN ANN	71	9	N/A ****	N/A
SHINTAWATI	2015	[34]	JAPAN	P	20	BONE SCAN	99mTc-MPD	BONENAVI BONE SCAN ANN	N/A	N/A	N/A	N/A
MITSUI	2012	[35]	JAPAN	R	42	BONE SCAN	99mTc-MDP	BONENAVI BONE SCAN ANN	73	8	65.3	N/A
UEMURA	2016	[36]	JAPAN	R	41	BONE SCAN	NOT SPECIFIED *	BONENAVI BONE SCAN ANN	73	N/A	56.8	N/A
UMEDA	2018	[37]	JAPAN	R	47	BONE SPECT/CT	99mTc-MDP	SPECT-BASED SEGMENTATION; BONENAVI BONE SCAN ANN	74	N/A	N/A	N/A
BROWN	2012	[38]	USA	R	20	BONE SCAN	99mTc-MDP	CAD ANALYSIS	N/A	N/A	N/A	N/A
MEIRELLES	2010	[39]	USA	P	39	BONE SCAN AND PET/CT	99mTc-HPD AND 18-F-FDG	EXINI BONE SCAN ANN	68	N/A	N/A	N/A
DENNIS	2012	[40]	USA	R	88	BONE SCAN	NOT SPECIFIED *	EXINI BONE SCAN ANN	67.7	8	95.95	N/A
REZA	2016	[41]	SWEDEN/UK/FINLAND/FRANCE	R	47	BONE SCAN	NOT SPECIFIED *	EXINI BONE SCAN ANN	68	N/A	83.1	N/A
FOSBØL	2018	[42]	DENMARK	R	88	BONE SCAN	NOT SPECIFIED *	EXINI BONE SCAN ANN	71	N/A	212	N/A
BLACKLEDGE	2014	[43]	UK	P	7	MRI	NONE	MARKOV RANDOM FIELD MODEL	N/A	N/A	N/A	N/A
PEREZ-LOPEZ	2016	[44]	UK	R	43	MRI AND BONE SCAN	NOT SPECIFIED *	MR SEGMENTATION AND EXINI BONE SCAN ANN	N/A	N/A	43	N/A
BRISSET	2015	[45]	USA/HOLLAND	P	12	CT AND MR	NONE	VOXEL-BASED ANALYSIS	N/A	N/A	N/A	N/A

LEGEND: R: retrospective; P: prospective; NS: non-specified; RW: review; N/A: not available; ANN: artificial neural network; * Unspecified 99mTc-labelled diphosphonate; ** Included a multicentric survey; *** Only median value was provided; **** Expressed as log.

**Table 2:** Advantages and disadvantages of the study types. The table includes also the relative frequency of the described method, when compared to the other ones.

Method	Advantages	Disadvantages	Relative Frequency
Neural network analysis applied to planar bone scan	- Wide diffusion of bone scan - Ease of use - Reproducibility - Prompt readability	- Overlap artifacts - Lack of specificity - Frequent need for manual corrections - Need for local databases - “Flare” responses	Common (prevalent diffusion of bone scan)
PET-based thresholding	- Relatively easy and prompt application - 3D volume definition - High specificity using co-registered CT	- Need for threshold recalibration - Need for active exclusion of non-bone and non-tumor uptakes	Uncommon
Hybrid CT- and PET/SPECT-based thresholding	- High accuracy thanks to dual segmentation - High information output suitable for ‘big data’ research applications	- Computationally intensive - Long elaboration times - Not yet validated for clinical practice	Rare (presently only research application)
MR-based and other non-isotopic methods	- Excellent lesion-to-background contrast - No radiation burden to the patient and to the general population	- Whole-body MRI still not diffusely utilized - Need for long acquisition and elaboration time	Rare (presently only research application)

## References

[1] Jemal A., Fedewa S.A., Ma J., Siegel R., Lin C.C., Brawley O., Ward E.M. (2015). Prostate Cancer Incidence and PSA Testing Patterns in Relation to USPSTF Screening Recommendations. JAMA.

[2] Heidenreich A., Bastian P.J., Bellmunt J., Bolla M., Joniau S., van der Kwast T., Mason M., Matveev V., Wiegel T., Zattoni F. (2014). EAU guidelines on prostate cancer. Part II: Treatment of advanced, relapsing, and castration-resistant prostate cancer. Eur. Urol..

[3] Quayle L., Ottewell P.D., Holen I. (2015). Bone Metastasis: Molecular Mechanisms Implicated in Tumour Cell Dormancy in Breast and Prostate Cancer. Curr. Cancer Drug Targets.

[4] Loriot Y., Bianchini D., Ileana E., Sandhu S., Patrikidou A., Pezaro C., Albiges L., Attard G., Fizazi K., De Bono J.S. (2013). Antitumour activity of abiraterone acetate against metastatic castration-resistant prostate cancer progressing after docetaxel and enzalutamide (MDV3100). Ann. Oncol..

[5] Kantoff P.W., Higano C.S., Shore N.D., Berger E.R., Small E.J., Penson D.F., Redfern C.H., Ferrari A.C., Dreicer R., Sims R.B. (2010). Sipuleucel-T immunotherapy for castration-resistant prostate cancer. N. Engl. J. Med..

[6] Berthold D.R., Pond G.R., Soban F., de Wit R., Eisenberger M., Tannock I.F. (2008). Docetaxel plus prednisone or mitoxantrone plus prednisone for advanced prostate cancer: Updated survival in the TAX 327 study. J. Clin. Oncol..

[7] Parker C., Nilsson S., Heinrich D., Helle S.I., O’Sullivan J.M., Fossa S.D., Chodacki A., Wiechno P., Logue J., Seke M. (2013). Alpha emitter radium-223 and survival in metastatic prostate cancer. N. Engl. J. Med..

[8] Omlin A., Pezaro C., Mukherji D., Mulick Cassidy A., Sandhu S., Bianchini D., Olmos D., Ferraldeschi R., Maier G., Thompson E. (2013). Improved survival in a cohort of trial participants with metastatic castration-resistant prostate cancer demonstrates the need for updated prognostic nomograms. Eur. Urol..

[9] Fizazi K., Massard C., Smith M., Rader M., Brown J., Milecki P., Shore N., Oudard S., Karsh L., Carducci M. (2015). Bone-related Parameters are the Main Prognostic Factors for Overall Survival in Men with Bone Metastases from Castration-resistant Prostate Cancer. Eur. Urol..

[10] Even-Sapir E., Metser U., Mishani E., Lievshitz G., Lerman H., Leibovitch I. (2006). The detection of bone metastases in patients with high-risk prostate cancer: 99mTc-MDP Planar bone scintigraphy, single- and multi-field-of-view SPECT, 18F-fluoride PET, and 18F-fluoride PET/CT. J. Nucl. Med..

[11] Nakajima K., Edenbrandt L., Mizokami A. (2017). Bone scan index: A new biomarker of bone metastasis in patients with prostate cancer. Int. J. Urol..

[12] Kwee S.A., Lim J., Watanabe A., Kromer-Baker K., Coel M.N. (2014). Prognosis Related to Metastatic Burden Measured by (1)(8)F-Fluorocholine PET/CT in Castration-Resistant Prostate Cancer. J. Nucl. Med..

[13] Yip S., Jeraj R. (2014). Use of articulated registration for response assessment of individual metastatic bone lesions. Phys. Med. Biol..

[14] Etchebehere E.C., Araujo J.C., Fox P.S., Swanston N.M., Macapinlac H.A., Rohren E.M. (2015). Prognostic Factors in Patients Treated with 223Ra: The Role of Skeletal Tumor Burden on Baseline 18F-Fluoride PET/CT in Predicting Overall Survival. J. Nucl. Med..

[15] Rohren E.M., Etchebehere E.C., Araujo J.C., Hobbs B.P., Swanston N.M., Everding M., Moody T., Macapinlac H.A. (2015). Determination of Skeletal Tumor Burden on 18F-Fluoride PET/CT. J. Nucl. Med..

[16] Lin C., Bradshaw T., Perk T., Harmon S., Eickhoff J., Jallow N., Choyke P.L., Dahut W.L., Larson S., Humm J.L. (2016). Repeatability of Quantitative 18F-NaF PET: A Multicenter Study. J. Nucl. Med..

[17] Harmon S.A., Perk T., Lin C., Eickhoff J., Choyke P.L., Dahut W.L., Apolo A.B., Humm J.L., Larson S.M., Morris M.J. (2017). Quantitative Assessment of Early [^18^F]Sodium Fluoride Positron Emission Tomography/Computed Tomography Response to Treatment in Men With Metastatic Prostate Cancer to Bone. J. Clin. Oncol..

[18] Etchebehere E.C., Araujo J.C., Milton D.R., Erwin W.D., Wendt R.E., Swanston N.M., Fox P., Macapinlac H.A., Rohren E.M. (2016). Skeletal Tumor Burden on Baseline 18F-Fluoride PET/CT Predicts Bone Marrow Failure After 223Ra Therapy. Clin. Nucl. Med..

[19] Lee J., Sato M.M., Coel M.N., Lee K.H., Kwee S.A. (2016). Prediction of PSA Progression in Castration-Resistant Prostate Cancer Based on Treatment-Associated Change in Tumor Burden Quantified by 18F-Fluorocholine PET/CT. J. Nucl. Med..

[20] Alva A., Nordquist L., Daignault S., George S., Ramos J., Albany C., Isharwal S., McDonald M., Campbell G., Danchaivijitr P. (2017). Clinical Correlates of Benefit From Radium-223 Therapy in Metastatic Castration Resistant Prostate Cancer. Prostate.

[21] Anand A., Morris M.J., Larson S.M., Minarik D., Josefsson A., Helgstrand J.T., Oturai P.S., Edenbrandt L., Roder M.A., Bjartell A. (2016). Automated Bone Scan Index as a quantitative imaging biomarker in metastatic castration-resistant prostate cancer patients being treated with enzalutamide. EJNMMI Res..

[22] Armstrong A.J., Kaboteh R., Carducci M.A., Damber J.E., Stadler W.M., Hansen M., Edenbrandt L., Forsberg G., Nordle O., Pili R. (2014). Assessment of the bone scan index in a randomized placebo-controlled trial of tasquinimod in men with metastatic castration-resistant prostate cancer (mCRPC). Urol. Oncol..

[23] Bieth M., Kronke M., Tauber R., Dahlbender M., Retz M., Nekolla S.G., Menze B., Maurer T., Eiber M., Schwaiger M. (2017). Exploring New Multimodal Quantitative Imaging Indices for the Assessment of Osseous Tumor Burden in Prostate Cancer Using (68)Ga-PSMA PET/CT. J. Nucl. Med..

[24] Schmuck S., von Klot C.A., Henkenberens C., Sohns J.M., Christiansen H., Wester H.J., Ross T.L., Bengel F.M., Derlin T. (2017). Initial Experience with Volumetric ^68^Ga-PSMA I&T PET/CT for Assessment of Whole-Body Tumor Burden as a Quantitative Imaging Biomarker in Patients with Prostate Cancer. J. Nucl. Med..

[25] Thomas L., Balmus C., Ahmadzadehfar H., Essler M., Strunk H., Bundschuh R.A. (2017). Assessment of Bone Metastases in Patients with Prostate Cancer-A Comparison between ^99m^Tc-Bone-Scintigraphy and [^68^Ga]Ga-PSMA PET/CT. Pharmaceuticals.

[26] Fiz F., Sahbai S., Campi C., Weissinger M., Dittmann H., Marini C., Piana M., Sambuceti G., la Fougere C. (2017). Tumor Burden and Intraosseous Metabolic Activity as Predictors of Bone Marrow Failure during Radioisotope Therapy in Metastasized Prostate Cancer Patients. Biomed. Res. Int..

[27] Miederer M., Thomas C., Beck J., Hampel C., Krieger C., Baque P.E., Helisch A., Schreckenberger M. (2015). Haematopoietic toxicity of radium-223 in patients with high skeletal tumour burden. Nuklearmedizin.

[28] Sadik M., Suurkula M., Hoglund P., Jarund A., Edenbrandt L. (2009). Improved classifications of planar whole-body bone scans using a computer-assisted diagnosis system: A multicenter, multiple-reader, multiple-case study. J. Nucl. Med..

[29] Lindgren Belal S., Sadik M., Kaboteh R., Hasani N., Enqvist O., Svarm L., Kahl F., Simonsen J., Poulsen M.H., Ohlsson M. (2017). 3D skeletal uptake of ^18^F sodium fluoride in PET/CT images is associated with overall survival in patients with prostate cancer. EJNMMI Res..

[30] Wassberg C., Lubberink M., Sorensen J., Johansson S. (2017). Repeatability of quantitative parameters of 18F-fluoride PET/CT and biochemical tumour and specific bone remodelling markers in prostate cancer bone metastases. EJNMMI Res..

[31] Kaboteh R., Gjertsson P., Leek H., Lomsky M., Ohlsson M., Sjostrand K., Edenbrandt L. (2013). Progression of bone metastases in patients with prostate cancer—Automated detection of new lesions and calculation of bone scan index. EJNMMI Res..

[32] Takahashi Y., Yoshimura M., Suzuki K., Hashimoto T., Hirose H., Uchida K., Inoue S., Koizumi K., Tokuuye K. (2012). Assessment of bone scans in advanced prostate carcinoma using fully automated and semi-automated bone scan index methods. Ann. Nucl. Med..

[33] Wakabayashi H., Nakajima K., Mizokami A., Namiki M., Inaki A., Taki J., Kinuya S. (2013). Bone scintigraphy as a new imaging biomarker: The relationship between bone scan index and bone metabolic markers in prostate cancer patients with bone metastases. Ann. Nucl. Med..

[34] Shintawati R., Achmad A., Higuchi T., Shimada H., Hirasawa H., Arisaka Y., Takahashi A., Nakajima T., Tsushima Y. (2015). Evaluation of bone scan index change over time on automated calculation in bone scintigraphy. Ann. Nucl. Med..

[35] Mitsui Y., Shiina H., Yamamoto Y., Haramoto M., Arichi N., Yasumoto H., Kitagaki H., Igawa M. (2012). Prediction of survival benefit using an automated bone scan index in patients with castration-resistant prostate cancer. BJU Int..

[36] Uemura K., Miyoshi Y., Kawahara T., Yoneyama S., Hattori Y., Teranishi J., Kondo K., Moriyama M., Takebayashi S., Yokomizo Y. (2016). Prognostic value of a computer-aided diagnosis system involving bone scans among men treated with docetaxel for metastatic castration-resistant prostate cancer. BMC Cancer.

[37] Umeda T., Koizumi M., Fukai S., Miyaji N., Motegi K., Nakazawa S., Takiguchi T. (2018). Evaluation of bone metastatic burden by bone SPECT/CT in metastatic prostate cancer patients: Defining threshold value for total bone uptake and assessment in radium-223 treated patients. Ann. Nucl. Med..

[38] Brown M.S., Chu G.H., Kim H.J., Allen-Auerbach M., Poon C., Bridges J., Vidovic A., Ramakrishna B., Ho J., Morris M.J. (2012). Computer-aided quantitative bone scan assessment of prostate cancer treatment response. Nucl. Med. Commun..

[39] Meirelles G.S., Schoder H., Ravizzini G.C., Gonen M., Fox J.J., Humm J., Morris M.J., Scher H.I., Larson S.M. (2010). Prognostic value of baseline [^18F^] fluorodeoxyglucose positron emission tomography and ^99m^Tc-MDP bone scan in progressing metastatic prostate cancer. Clin. Cancer Res..

[40] Dennis E.R., Jia X., Mezheritskiy I.S., Stephenson R.D., Schoder H., Fox J.J., Heller G., Scher H.I., Larson S.M., Morris M.J. (2012). Bone scan index: A quantitative treatment response biomarker for castration-resistant metastatic prostate cancer. J. Clin. Oncol..

[41] Reza M., Jones R., Aspegren J., Massard C., Mattila L., Mustonen M., Wollmer P., Tragardh E., Bondesson E., Edenbrandt L. (2016). Bone Scan Index and Progression-free Survival Data for Progressive Metastatic Castration-resistant Prostate Cancer Patients Who Received ODM-201 in the ARADES Multicentre Study. Eur. Urol. Focus.

[42] Fosbol M.O., Petersen P.M., Kjaer A., Mortensen J. (2018). ^223^Ra Therapy of Advanced Metastatic Castration-Resistant Prostate Cancer: Quantitative Assessment of Skeletal Tumor Burden for Prognostication of Clinical Outcome and Hematologic Toxicity. J. Nucl. Med..

[43] Blackledge M.D., Collins D.J., Tunariu N., Orton M.R., Padhani A.R., Leach M.O., Koh D.M. (2014). Assessment of treatment response by total tumor volume and global apparent diffusion coefficient using diffusion-weighted MRI in patients with metastatic bone disease: A feasibility study. PLoS ONE.

[44] Perez-Lopez R., Lorente D., Blackledge M.D., Collins D.J., Mateo J., Bianchini D., Omlin A., Zivi A., Leach M.O., de Bono J.S. (2016). Volume of Bone Metastasis Assessed with Whole-Body Diffusion-weighted Imaging Is Associated with Overall Survival in Metastatic Castration-resistant Prostate Cancer. Radiology.

[45] Brisset J.C., Hoff B.A., Chenevert T.L., Jacobson J.A., Boes J.L., Galban S., Rehemtulla A., Johnson T.D., Pienta K.J., Galban C.J. (2015). Integrated multimodal imaging of dynamic bone-tumor alterations associated with metastatic prostate cancer. PLoS ONE.

[46] Bastawrous S., Bhargava P., Behnia F., Djang D.S., Haseley D.R. (2014). Newer PET application with an old tracer: Role of 18F-NaF skeletal PET/CT in oncologic practice. Radiographics.

[47] Eder M., Schafer M., Bauder-Wust U., Hull W.E., Wangler C., Mier W., Haberkorn U., Eisenhut M. (2012). ^68^Ga-complex lipophilicity and the targeting property of a urea-based PSMA inhibitor for PET imaging. Bioconjug. Chem..

[48] Ryan C.J., Shah S., Efstathiou E., Smith M.R., Taplin M.E., Bubley G.J., Logothetis C.J., Kheoh T., Kilian C., Haqq C.M. (2011). Phase II study of abiraterone acetate in chemotherapy-naive metastatic castration-resistant prostate cancer displaying bone flare discordant with serologic response. Clin. Cancer Res..

[49] Matheoud R., Goertzen A.L., Vigna L., Ducharme J., Sacchetti G., Brambilla M. (2012). Five-year experience of quality control for a 3D LSO-based whole-body PET scanner: Results and considerations. Phys. Med..

[50] Oldan J.D., Hawkins A.S., Chin B.B. (2016). ^18^F Sodium Fluoride PET/CT in Patients with Prostate Cancer: Quantification of Normal Tissues, Benign Degenerative Lesions, and Malignant Lesions. World J. Nucl. Med..

[51] Sambuceti G., Brignone M., Marini C., Massollo M., Fiz F., Morbelli S., Buschiazzo A., Campi C., Piva R., Massone A.M. (2012). Estimating the whole bone-marrow asset in humans by a computational approach to integrated PET/CT imaging. Eur. J. Nucl. Med. Mol. Imaging.

[52] Fiz F., Marini C., Piva R., Miglino M., Massollo M., Bongioanni F., Morbelli S., Bottoni G., Campi C., Bacigalupo A. (2014). Adult advanced chronic lymphocytic leukemia: Computational analysis of whole-body CT documents a bone structure alteration. Radiology.

[53] Fiz F., Marini C., Campi C., Massone A.M., Podesta M., Bottoni G., Piva R., Bongioanni F., Bacigalupo A., Piana M. (2015). Allogeneic cell transplant expands bone marrow distribution by colonizing previously abandoned areas: An FDG PET/CT analysis. Blood.

[54] Marini C., Bruno S., Fiz F., Campi C., Piva R., Cutrona G., Matis S., Nieri A., Miglino M., Ibatici A. (2017). Functional Activation of Osteoclast Commitment in Chronic Lymphocytic Leukaemia: A Possible Role for RANK/RANKL Pathway. Sci. Rep..

